# Container Closure and Delivery Considerations for Intravitreal Drug Administration

**DOI:** 10.1208/s12249-021-01949-4

**Published:** 2021-03-11

**Authors:** Ashwin C. Parenky, Saurabh Wadhwa, Hunter H. Chen, Amardeep S. Bhalla, Kenneth S. Graham, Mohammed Shameem

**Affiliations:** Formulations Development, Regeneron Pharmaceuticals, Inc., 777 Old Saw Mill River Road Tarrytown, New York, 10591 USA

**Keywords:** Ocular, Syringe, Needle, Sterilization, Formulation

## Abstract

Intravitreal (IVT) administration of therapeutics is the standard of care for treatment of back-of-eye disorders. Although a common procedure performed by retinal specialists, IVT administration is associated with unique challenges related to drug product, device and the procedure, which may result in adverse events. Container closure configuration plays a crucial role in maintaining product stability, safety, and efficacy for the intended shelf-life. Careful design of primary container configuration is also important to accurately deliver small volumes (10-100 μL). Over- or under-dosing may lead to undesired adverse events or lack of efficacy resulting in unpredictable and variable clinical responses. IVT drug products have been traditionally presented in glass vials. However, pre-filled syringes offer a more convenient administration option by reducing the number of steps required for dose preparation there by potentially reducing the time demand on the healthcare providers. In addition to primary container selection, product development studies should focus on, among other things, primary container component characterization, material compatibility with the formulation, formulation stability, fill volume determination, extractables/leachables, and terminal sterilization. Ancillary components such as disposable syringes and needles must be carefully selected, and a detailed administration procedure that includes dosing instructions is required to ensure successful administration of the product. Despite significant efforts in improving the drug product and administration procedures, ocular safety concerns such as endophthalmitis, increased intraocular pressure, and presence of silicone floaters have been reported. A systematic review of available literature on container closure and devices for IVT administration can help guide successful product development.

## INTRODUCTION

**Fig. 1:**
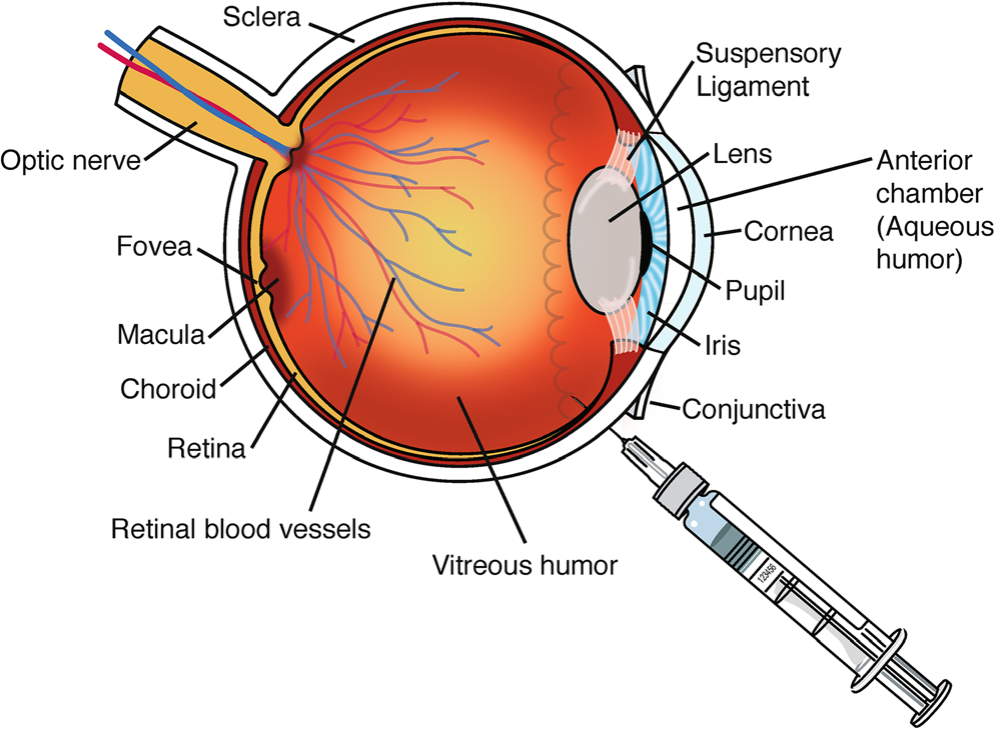
Illustration of the ocular anatomy and intravitreal injection for the treatment of ocular diseases

Intravitreal (IVT) administration is currently the standard of care for administration of anti-VEGF agents to treat wet age-related macular degeneration (wet AMD). In addition to wet AMD, IVT injections are also administered to treat ocular conditions such as branched and central vein occlusion, diabetic macular edema (DME), and uveitis. Since the 1990s, approval of intravitreal drug products to manage and treat retinal diseases has experienced significant and rapid growth. As of 2020, there are a total of thirteen drug products approved for IVT administration in the USA. These products include anti-VEGF agents, a synthetic corticosteroid, a proteolytic enzyme, and long-acting delivery systems. It is estimated that around 5.9 million intravitreal injections were administered in 2016 alone. The rapid growth in IVT administration of drug products could be attributed to intense research efforts in identifying targets that specifically treat and manage diseases in addition to significant improvements in delivery systems and devices, administration procedures, relevant materials, and formulations that help preserve the safety and efficacy of therapeutics (1). Figure 1 is an illustration of an IVT injection and anatomy of the human eye (not drawn to scale).

The development of drug products for IVT administration is particularly challenging as ancillary injection components (such as syringes and needles routinely used for IVT injections) were not developed specifically for IVT administration in the eye and even today, there is considerable reliance on devices that are used for delivering non-ophthalmic therapeutics (2). Historically, it was believed that the ocular tissue is an “immune privileged” site owing to the blood-retinal barrier, blood-aqueous barrier, and tight junctions that restrict entry of cells, proteins, and lipids from the systemic circulation into ocular tissues. However, significant advances have been made in understanding the ocular cellular and molecular mechanisms such as the anterior chamber–associated immune deviation (ACAID). The mechanism of ACAID suggests that ocular antigen-presenting cells can transit to the spleen to initiate an antigen specific “regulatory” immune response. Such mechanisms highlight the presence of cell-mediated immune responses present in the eye that can lead to a humoral response to the antigen injected into the eye. Therefore, incidence of ocular inflammation, ocular tissue damage, and anti-drug antibody responses (ADA) are clinical responses that are monitored during the development of biotherapeutics specifically for ocular indications (3). Hence, careful and detailed assessment of the drug product and device evaluation is critical for the development of successful IVT therapeutics.

## ADMINISTRATION OF IVT INJECTIONS

IVT administration is now commonplace which has made the administration procedure well established. However, successful IVT injections depend on several important considerations before, during, and after the procedure has been performed. Briefly, considerations such as dose preparation procedure, injection environment, application of local anesthesia to the ocular tissue, use of adequate personal protective equipment, disinfection of the ocular surface, and use of topical antibiotics are critical for ensuring patient safety following IVT administration.

### Dose Preparation Procedure

#### Vials

Single-use vial drug product presentations involve more preparation steps as compared to a pre-filled syringe. Although detailed instructions are provided in respective drug product labels, a typical procedure is outlined here. The plastic cap on the drug product vial is removed and the top of the vial is wiped with an alcohol wipe. A filter needle (e.g., 19-gauge × 1½-in., 5-μm) is attached to a syringe and the formulation is drawn aseptically into the syringe from the vial. The plunger rod is pulled back sufficiently to ensure the formulation is in the syringe completely before removing the filter needle and replacing it with a needle which will be used for the IVT injection (e.g., 30-gauge × ½-in.). To ensure the dose to be administered is accurate, the syringe is held upright and gently tapped to release any air bubbles and the plunger is pushed to expel the drug until the plunger tip aligns with the appropriate dose volume for administration (typically 0.050 mL).

#### Prefilled Syringe

Pre-filled syringes have a relatively less cumbersome method of dose preparation as the product is supplied in the syringe. In brief, the pre-filled syringe cap is removed from the syringe and the injection needle for administration of the product to the patient is attached (e.g., 30-gauge × ½-in.). The syringe is held upright, checked for air bubbles, and gently tapped to remove the bubbles which rise to the top. The bubbles are expelled with the excess drug and dose adjusted by aligning the plunger to the dose mark as mentioned in the product label.

### IVT Injection Procedure

Injection site for IVT administration is generally performed between the vertical and horizontal rectus muscles at the pars plana, 3.5–4.0 mm posterior to the limbus as a perpendicular injection (4). Injection procedures require utmost skill, precision, and experience. A survey of retina specialists across the USA revealed that most of the specialists did not measure the distance from the limbus on injection while predominantly injecting in the inferior temporal quadrant (5). Further detailed discussion on IVT administration procedure is beyond the scope of this manuscript.

## NEEDLES AND SYRINGES AS ANCILLARY COMPONENTS FOR ADMINISTRATION OF IVT PRODUCTS

### Clinical Impact of Needle Gauge Used for IVT Injections

In addition to the administration procedure for IVT injections, other important considerations when performing IVT injections include the selection of administration components and devices that will result in safe and effective dosing of the therapeutic. Recommended needle gauges packaged with IVT drug products approved by the FDA are listed in Tables I and II. Vitravene® (fomivirsen sodium) was the first US FDA-approved IVT oligonucleotide product used in patients suffering from cytomegalovirus (CMV) retinitis. Approved in 1998, fomivirsen sodium was administered using a low-volume syringe (e.g., tuberculin) and a 30G needle at an injection volume of 0.050 mL/dose (6).

**Table I:** List of Approved IVT Implants in the USA

Product name	Molecule	Owner/company	Dose/duration	Needle gauge	Dimensions	References
ILUVIEN^®^ ocular implant	Fluocinolone acetonide	Alimera Sciences, Inc.	0.19 mg	25 gauge	3.5 mm × 0.37 mm	(7)
OZURDEX^®^ ocular implant	Dexamethasone	Allergan, Inc.	0.7 mg	22 gauge	0.46 mm × 6 mm	(8,9)
RETISERT^®^ ocular implant	Fluocinolone acetonide	Bausch & Lomb Inc.	0.59 mg/30 months	NA (sutured)	3 mm × 2 mm × 5 mm	(10)
YUTIQ^™^ implant	Fluocinolone acetonide	EyePoint Pharmaceuticals US, Inc.	0.18 mg	25 gauge	3.5 mm × 0.37 mm	(11)

*VITRASERT® not included as it was discontinued*

**Table II:** Approved IVT Liquid/Suspension Drug Product Formulations

Product name	Molecule	Manufacturer	Formulation/composition	Primary container closure	Needle gauge	Reference
LUCENTIS^®^ injection solution	Ranibizumab	Genentech, Inc.	6 mg/mL or 10 mg/mL LUCENTIS® aqueous solution with 10 mM histidine HCl, 10% α, α-trehalose dihydrate, 0.01% polysorbate 20, pH 5.5	Vial	30G	(12)
LUCENTIS^®^ pre-filled syringe	Ranibizumab	Genentech, Inc.	6 mg/mL or 10 mg/mL LUCENTIS® (0.5 mg dose prefilled syringe) aqueous solution with 10 mM histidine HCl, 10% α, α-trehalose dihydrate, 0.01% polysorbate 20, pH 5.5	Pre-filled syringe	30G	(12)
EYLEA^®^ injection solution	Aflibercept	Regeneron Pharmaceuticals, Inc.	40 mg/mL in 10 mM sodium phosphate, 40 mM sodium chloride, 0.03% polysorbate 20, and 5% sucrose, pH 6.2	Vial	30G	(13)
EYLEA® pre-filled syringe	Aflibercept	Regeneron Pharmaceuticals, Inc.	40 mg/mL in 10 mM sodium phosphate, 40 mM sodium chloride, 0.03% polysorbate 20, and 5% sucrose, pH 6.2	Pre-filled syringe	30G	(13)
BEOVU^®^ injection solution	Brolucizumab-dbll	Novartis AG	6 mg brolucizumab-dbll, polysorbate 80 (0.02%), sodium citrate (10 mM), sucrose (5.8%), and Water for Injection, USP and with a pH of approximately 7.2	Vial	30G	(14)
MACUGEN^®^ pre-filled syringe	Pegaptanib sodium	Gilead Sciences, Inc.	3.47 mg/mL pegaptanib sodium chloride, monobasic sodium phosphate monohydrate, dibasic sodium phosphate heptahydrate, hydrochloric acid, and/or sodium hydroxide	Pre-filled syringe	30G	(15)
JETREA^®^ injection solution	Ocriplasmin	Oxurion NV	0.5 mg ocriplasmin in 0.2 mL citric-buffered solution (2.5 mg/mL)	Vial	30G	(16)
TRIESENCE^®^	Triamcinolone acetonide	Alcon Laboratories, Inc.	40 mg of triamcinolone acetonide, with sodium chloride for isotonicity, 0.5% (w/v) carboxymethylcellulose sodium and 0.015% polysorbate 80	Vial	27G	(17)
TRIVARIS®	Triamcinolone acetonide	Allergan, Inc.	8 mg triamcinolone acetonide in 0.1 mL (8% suspension) in a vehicle containing w/w percents of 2.3% sodium hyaluronate; 0.63% sodium chloride; 0.3% sodium phosphate, dibasic; 0.04% sodium phosphate, monobasic; and water for injection, pH 7.0 to 7.4	Vial	27G	(18)

Patients experience pain on IVT injections due to the presence of pressure or sensory receptors on the sclera, episclera, conjunctiva, or changes in intraocular pressure (IOP). Factors that may contribute to pain on injection include the drug/solution, rate of injection, volume injected, size/form of the injected product, the needle characteristics (bevel design and gauge), and the injection technique. As of 2020, half-inch needles between 27G and 30G have been used for IVT injections, except for OZURDEX®, an IVT sustained release implant containing dexamethasone which utilizes a 22G needle (1). Chaturvedi *et al.* published on administration procedure from 281 retinal specialists across the USA that focused on pre-administration, administration, and post-administration IVT procedures. Statistical analysis on the injection procedure revealed that about 61% (170/279) of retinal specialists use a 30-gauge needle while 21% (59/279) of retinal specialists chose to use the 31-gauge needle (5). Rodrigues *et al.* performed studies to determine the impact of needle gauge on vitreal reflux and pain on injection. It was observed that the force required to penetrate the sclera using a 27G needle was twice as much compared to a 31G needle. Results also demonstrate a significant reduction in pain on injection and vitreal reflux when 29G and 30G needles are used in comparison to 26G or 27G needle (19). Vitreal reflux has been identified as a potential complication for IVT injection as it may be associated with loss of injected drug from the vitreous and adverse events like endophthalmitis. There have been several clinical studies investigating the impact of needle sizes on vitreal reflux and IOP after IVT injection. Muto and Machida reported similar rates of vitreal reflux with 30G needle and 32G needle in patients receiving aflibercept for the first time (20). However, it was observed that immediate post-injection IOP was higher when 30G needles were used as opposed to 32G needles (20). In addition to needle gauge, other important factors such as needle geometry, syringe size, backpressure at the injection site, formulation characteristics, and behavior (Newtonian/non-Newtonian) play a significant role in determining the injection forces.

### Clinical Experience with Advanced Needle Geometry

#### Thin-Wall Needles and Micro-tapered Needles

**Fig. 2:**
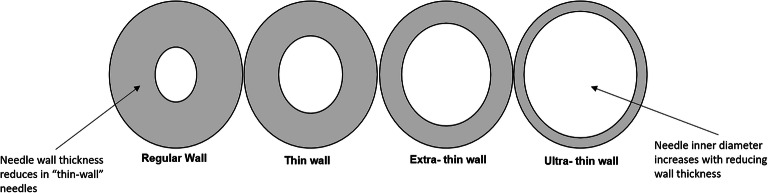
Illustration of a cross-sectional area of a needle. Wall thickness and inner diameter for varying needle types from regular wall, thin wall, extra-thin wall, and ultra-thin wall. (Not drawn to scale)

The choice of needles arises from considerations such as allowable injection force, site of administration, and clinical experience with certain needle gauges and lengths. In recent years, manufacturers have the design capability to alter the needle inner diameter to significantly influence the injection force required to administer biologics. International Standards Organization (ISO) 9626:2016, provides needle dimension details, as mentioned in Table III, to manufacturers as target dimensions for manufacturing needles. A clinical trial investigated the impact of extra-thin-wall needles (31G and 32G) on self-administered subcutaneous (SC) injections using three different insulin pens. It was observed that patients significantly preferred extra-thin-wall needles compared to thin-wall or regular-wall needles. The study demonstrated extra-thin-wall needles improved flow characteristics and pressure required to inject insulin, which corresponds to lower injection force, reduced time to inject, and greater confidence in completing the patient-administered SC injection (21). Therefore, extra-/ultra-thin-wall needles for a given needle gauge may translate to improved patient experience and injection forces for intravitreal administration as well. Figure 2 demonstrates the various needle geometries for any given needle gauge.

**Table III:** Needle Gauge Dimensions from ISO 9626:2016 Standards (Minimum inner diameters)

Needle gauge	Regular-wall inner diameter (mm)	Thin-wall inner diameter (mm)	Extra-thin-wall inner diameter (mm)	Ultra-thin-wall inner diameter (mm)
30	0.133	0.165	0.190	0.240
32	0.089	0.105	0.125	0.146

^©^ISO. This material is reproduced from ISO 9626:2016, with permission of the American National Standards Institute (ANSI) on behalf of the International Organization for Standardization. All rights reserved

Needle geometry and sharpness are important considerations for improved patient experience and injection performance during injection procedures. Injection performance of various needles was evaluated in a clinical study examining self-injection of insulin subcutaneously, which concluded that even though injection forces in 28G to 33G micro-tapered needles (Terumo Corporation, Tokyo, Japan) were similar to the standard 31G thin-wall (TW) needles (Becton Dickinson), patients concerned about pain often preferred micro-tapered needle over TW needles. The 28G to 33G micro-tapered needles have an advantage when injecting non-Newtonian fluids as the taper allows for higher shear forces thereby reducing the resistance during injection. Therefore, micro-tapered needles have the potential to reduce pain and discomfort of injections as compared to traditional needles. Krayukhina *et al.* demonstrated the advantage of using tapered needles as opposed to thin-wall needles concluding that the injection forces required for a 29G tapered needle was consistently lower than a 29G thin-wall needle when injecting polyethylene glycol 3350, carboxymethyl cellulose, etanercept, and omalizumab solutions. Interestingly, injection forces for the 29G tapered needle for glycerin, polyethylene glycol 3350, etanercept, and 70 mg/mL omalizumab solution were similar to a 27G thin-wall needle. In the case of non-Newtonian solutions such as 125 mg/mL omalizumab and carboxymethyl cellulose, injection forces required for the 29G tapered needle were significantly lower than the 27G thin-wall needle (22). Figure 2 is an illustration of the various types of needles with increasing inner diameters (not drawn to scale).

#### Needle Bevel Designs

**Fig. 3:**
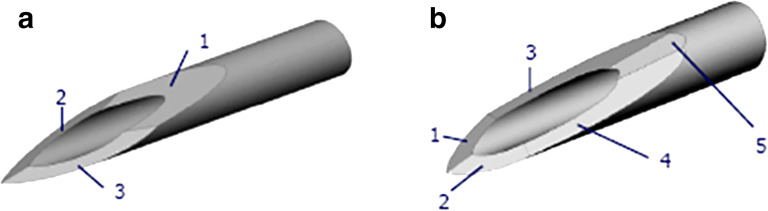
Illustration of 3-bevel design (**a**) and 5-bevel design (**b**) for needles. This image has been reproduced from Hirsch *et al.* (2012) (#54) with permission from SAGE Publications

Needle tip geometry and bevel designs such as the number of bevels, angularity, and tip facets can impact needle insertion forces and perceived pain in patients (23). When 31G and 32G needles with a 5-bevel tip design were tested against similar needles with 3-bevel tip designs, significant reduction in insertion forces was documented for the 5-bevel tip based on patient’s experience when injecting interferon and insulin (24) (23). During *in vitro* testing on human skin substitute, the 5-bevel tip was observed to reduce penetration force by 23% on average compared to a 3-bevel needle tip. In a blinded study performed on diabetic patients comparing the 5-bevel design and 3-bevel design, patients overwhelmingly rated the 5-bevel design significantly more comfortable, easier to insert, and preferable than the 3-bevel needle tip (23). These results indicate that investigating bevel designs for IVT injection needles may result in the selection of components that improve patient experience (Fig. 3).

### Syringes Used for IVT Injections

Intravitreal injections have become increasingly common and the standard of care for retinal diseases which makes syringes indispensable for the treatment of retinal diseases. For drug products where the primary container is a vial, syringes are an ancillary component used to draw the product from the vial and inject into the vitreous, as described in previous sections. Syringes are generally made of materials such as polypropylene, polycarbonate, or glass. Commonly used syringes for IVT injections are 1-mL luer-lock and slip-luer syringes with 0.5-mL or 0.3-mL syringes being used less often. Generally, syringes and needles are two separate components unless the use of staked-in needle syringes is desired (2). Scott *et al.* studied the clinical impact of glass syringes with a slip-luer design and staked needle design on the presence of silicone oil floaters in the vitreous cavity post IVT injection. Patients who received IVT triamcinolone with a staked needle syringe had significantly higher rates of floaters as compared to patients receiving triamcinolone with a slip-luer syringe (25). Although labels for IVT drug products packaged in vials may not mention specific syringes for IVT injections, retinal specialists and drug development scientists should consider these observations to minimize any risk of unwanted clinical events. With increasingly global outreach of therapeutics and inconsistencies in IVT dose preparation practices, it would be important for drug development scientists to consider minimizing inconsistencies and develop drug products that can be utilized with minimal steps for dose preparation and handling.

### Syringeability and Injectability: Impact of Syringe Size and Type on Injection Force

Syringeability and injectability for parenteral products are critical attributes that need to be considered during development of a drug product. Syringeability refers to the force required for an injectable therapeutic to easily pass through a hypodermic needle of predetermined gauge and length, at a specified injection rate. Injectability refers to the performance of the formulation, syringe, and needle during injection into target tissues. Factors that are considered while determining the syringeability of a drug product include ease of withdrawal, accuracy of dose measurements, clogging, and foaming, while injectability includes factors such as pressure or force required for injection, back pressure at the tissue site, and evenness of flow. In case of drug products manufactured in vials, assessment of both syringeability and injectability is necessary and both contribute to accurate dosing of patients in the clinic. For drug products in pre-filled syringes and autoinjectors, significant evaluation is done to assess the performance according to FDA Guidance for Industry on container closure systems while other guidance documents such as ICH Q6A provide guidance on test procedures, injectability, and functionality of the delivery system (26).

For a given formulation (assuming Newtonian behavior), injectability at a predetermined speed is governed by factors such as needle gauge and surface area of the syringe plunger. Pressure generated within the syringe barrel (*P*) during injection is directly proportional to the force (*F)* exerted on the back of the syringe plunger and inversely proportional to surface area of the syringe plunger (*A*) (Eq. 1). Therefore, in the case of certain high-viscosity formulations, one approach to reduce injection forces could entail the use of syringes with reduced barrel diameter which corresponds to lower surface area of the syringe plunger.

1 $$F=P\ast A$$

However, a more detailed equation known as the Hagen Poiseuille (Eq. 2) can be derived for estimation of syringe glide force which considers dimensions of the syringe, needle, flow rate, and viscosity of the fluid. In Eq. 2, *F* is the glide force (*N*), $\left(\frac{\delta v}{\delta t}\right)$ is the volumetric flow rate, μ is the fluid viscosity, *L* is the needle length, *R*_needle_ is the needle inner radius, *R*_syringe_ is the inner syringe barrel radius, and *F*_friction_ is the frictional force between the plunger and syringe barrel (27,28).

2 $$F=\frac{8\left(\frac{\delta v}{\delta t}\right)\ L\ {R}^2\mathrm{syringe}}{{{\mathrm{R}}^4}_{\mathrm{needle}}}\ast \mu +{F}_{\mathrm{Friction}}$$

Product development scientists have an opportunity to optimize the injection glide force with modulation of formulation composition in combination with device selection. Extensive characterization of rheological properties must be performed during product development. For solutions that exhibit non-Newtonian behavior, appropriate excipients must be screened to maintain acceptable viscosity profiles. Allmendinger *et al.* performed a detailed investigation to derive equations to predict injection forces for high-concentration protein formulations that exhibit non-Newtonian behavior. Viscosity measurements were performed at high shear rates on commercially available protein therapeutics, and a model was developed to understand the non-Newtonian behavior of shear-thinning formulations. The authors derived an equation based on the transformation of the Hagen-Poiseuille law into an equation that accounts for shear rate–dependent viscosity changes that occur when a non-Newtonian fluid travels through the needle (28).

3 $$F={2}^{n+2}\ {\uppi}^{1-n}\times L\times {R^2}_{\mathrm{syringe}}\times K\times {\left(\delta V/\delta t\right)}^n\times {R_{\mathrm{needle}}}^{\hbox{--} \left(3\mathrm{n}+1\right)}\times {\left(3n+1/2n+1\right)}^{n-1}+{F}_{\mathrm{friction};f\left(\updelta \mathrm{v}/\updelta \mathrm{t}\right)}$$$$

In Eq. 3, *F* is the syringe glide force, *K* is the flow consistency index (Pa s^n^ ), and *n* is the power law index (dimensionless). *K* can be derived from the Ostwald-de Waele equation where *n* < 1 shear thinning behavior, *n* = 1 Newtonian behavior, and *n* > 1 shear thickening behavior. *δV/δt* is the volumetric flow, *L* is the length of needle, *R*_needle_ is the needle inner radius, *R*_syringe_ is the inner syringe barrel radius, and *F*_friction_ is the frictional forces between the plunger and syringe barrel.

These equations take into account dimensions of the needle, syringe, and behavior of the formulation. Non-Newtonian behavior can be observed in high-concentration protein formulations and/or polymer formulations, and these equations can be used for practical applications where drug products can be improved for ease of use, safety, and efficacy with adequate understanding of factors that have the highest influence on injection force. From Eqs. 2 and 3, the highest-powered factors are radii of the syringe barrel and needle. Therefore, the development of combination drug products for a given administration route should consider using these equations to recommend a specific needle gauge, syringe, or device for evaluation.

#### Formulation Considerations for Development of Injectable Products

Drug products developed for intravitreal injections can either be stored in type I borosilicate glass vials or type I borosilicate glass pre-filled syringes as listed in Table II. For pre-filled syringe drug products, liquid formulations come in contact with the syringe barrel (interior), syringe plunger, and needle. Siliconization of the interior surface of the syringe barrel is performed to provide adequate lubrication between the glass syringe barrel and plunger interface. This helps in adequate functionality of the pre-filled syringe to maintain acceptable break-loose and glide force. However, with the presence of silicone in the internal surface of the syringe barrel, formulations can interact with the silicone which may impact product quality and syringeability. Formulation factors such as buffers and surfactants have been shown to significantly impact the functionality of syringes by changing the silicone oil coverage in the barrel and lubricity. For example, the type of surfactant used in the formulation can impact glide force for a pre-filled syringe. Wang *et al.* demonstrated a significant increase in syringe glide force when pre-filled syringes containing formulation with polysorbate 80 were incubated at 40°C. Interestingly, formulations with poloxamer 188 when stored at 40°C in pre-filled syringes did not exhibit an increase in glide force. Schlieren imaging of the syringes detected removal of the silicone oil layer in the polysorbate 80 syringes when compared to intact silicone oil layer in syringes containing formulations with poloxamer 188. The authors also discuss the correlation of surfactant hydrophilic-lipophilic balance (HLB) values and surface tension values with glide force for pre-filled syringe development (29). Similarly, buffers/tonicity agents and formulation pH can also impact the silicone oil lubrication in pre-filled syringes that may lead to changes in the syringe functionality during storage (30).

Formulation viscosity is directly correlated to injection force, increase in formulation viscosity can lead to increased injection force for a drug product, and this may also increase the injection time for a given injection volume. IVT drug products are generally restricted to less than 100 microliters per injection. Therefore, clinical studies that target high IVT doses of therapeutics may require high-concentration protein formulations. In general, viscosity for high-protein concentration formulations increase exponentially (>100 mg/mL) and therefore, scientists must account for the associated impact on injection glide force (31). In addition to considerations such as viscosity of the formulations at target concentrations, it is advisable to understand viscosity profiles of the protein formulations at the upper specification concentration as per manufacturing capability. Additional optimization in fill-finish operations may be required for solutions exhibiting non-Newtonian behavior (32). This would provide an overall understanding of injection glide force at protein concentrations relevant to real-world manufacturing capability for drug products (33). Another important consideration for developing IVT drug products is understanding the impact of temperature on syringeability of the drug product. Since most biologics are stored at 2–8°C, temperature of the drug product can alter viscosity profile of the protein formulation at a specified concentration. Generally, viscosity of a protein formulation increases with decrease in temperature (34). Therefore, it is important to understand the impact of temperature on viscosity of the IVT formulation and consider formulation optimization that account for incomplete equilibration of the drug product to room temperature which may influence viscosity and syringeability. Therefore, formulation factors, such as type of excipients, excipient concentration, buffers, formulation pH, and protein concentration, can play a significant role in syringeability of pre-filled syringe drug products.

#### Accuracy and Repeatability of Delivered Volume

**Fig. 4:**
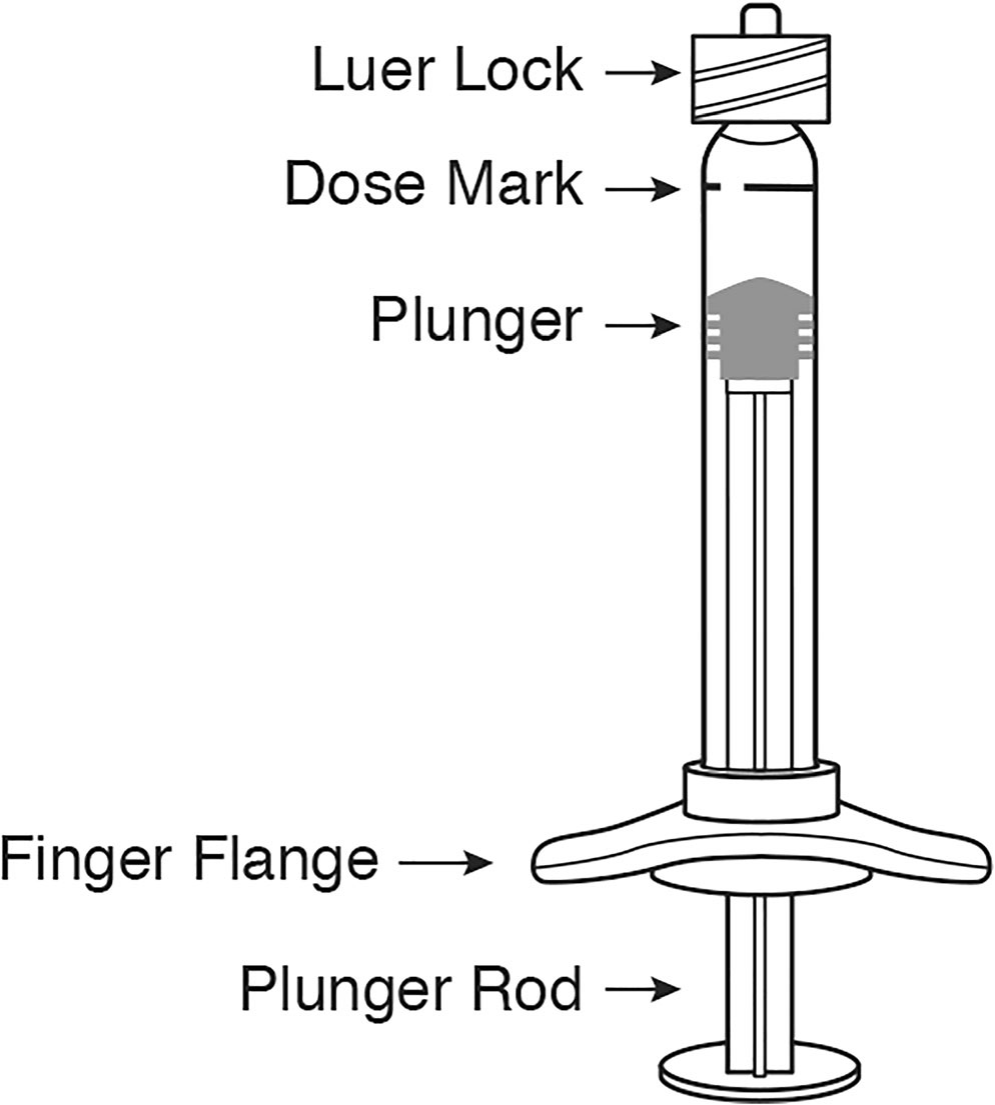
Illustration of a pre-filled syringe for intravitreal administration

Accuracy and repeatability of delivered/injected volume are critical to the efficacy and, in some cases, safety of a given drug. Therefore, it is essential that the proposed administration system/configuration is characterized for delivered volume during development. Delivering less than the target volume may lead to under dosing which will impact the efficacy and, in the case of some drugs, may impact the frequency of injection as well. Injection volume is usually based on the intended dose and concentration of a product. IVT injection volumes are typically between 0.05 and 0.1 mL. However, lower volumes in the range of 0.01–0.025 mL have been recently used to treat retinopathy of prematurity (ROP) in infants (35). Higher injection volumes (> 0.1 mL) may be associated with a transient increase in the intraocular pressure (IOP). Since many patients receiving IVT injections may already have impaired perfusion, an increase in IOP may further amplify the condition. IOP increase after IVT injection may also be dependent on factors such as the intraocular volume, scleral thickness, and scleral rigidity. Kotliar *et al.* studied the effect of 0.1 mL injection of triamcinolone on IOP in myopic, emmetropic, and hyperopic eyes (30). IOP increase of 40.6 ± 12.1 mm Hg was observed after injection compared to pre-operative values across different eyes. The increase in IOP was transient and returned to < 20 mmHg within 2 h. Eyes with shorter axial length showed a higher increase in IOP. It is also essential to minimize variability in injected volume at both lower and higher ends. During development, selection of delivery components that not only accurately deliver the desired target dose volume but also minimize variability in the delivered volume in clinical settings is desirable. The commercially available vial presentations IVT drug products usually have an overfill to account for volume losses during dose preparation. (36). The administering physician is typically expected to draw a volume larger than the dosing volume into a syringe through a larger gauge needle and expel excess volume through a narrower gauge injection needle to achieve the intended dose. The situation for DP manufactured in pre-filled syringes is similar. For example, one ranibizumab pre-filled syringe (PFS) contains 0.165 mL of drug to administer a 0.05-mL dose (36). In case of a pre-filled syringe, the excess volume contained in the syringe is expelled through the attached administration needle. The dose is subsequently set by aligning the plunger with the desired dose mark on the graduated syringe or in the case of pre-filled syringes, with a printed/labeled dose mark as illustrated in Fig. 4. Various studies have emphasized the need for special care in the selection of delivery component including the plunger, optimization of dead space or hold up volume, and selecting syringe size that is closer to the dose volume to ensure accuracy and repeatability. A study to determine the accuracy of IVT volume delivered using three different products found that 84% of the injections were greater and 16% of injections were less than the intended 0.05mL volume respectively (37). Another study showed that the use of a low dead space plunger increased precision while using a smaller size syringe (0.5 mL) resulted in higher accuracy compared to a 1-mL syringe when delivering a 0.05mL dose (38). It is important to note that due to the small intravitreal space and, therefore, a limited injection volume range, higher concentration formulations are necessary to deliver a higher dose. However, higher protein concentration may be associated with increased viscosity that may result in reduced accuracy of the delivered dose. Solutions with viscosity in the range of 1–80 cP were evaluated when delivering dose volumes in the range of 0.03-0.1mLusing various commercially available 1-mL disposable plastic syringes. Dose errors as high as 40% were observed for high-viscosity solutions (45 cP) depending on the type of syringe used (39).

For pre-filled syringes for intravitreal products, glass vials are routinely used for packaging of IVT products and require ancillary components such as syringes for administration as described above. However, in recent years, prefilled syringes (PFS) have become a preferred choice for parenteral biopharmaceutical products. PFSs have unique advantages such as convenience and safe handling (reduced potential for needle-stick injury) compared to conventional vial drug products. Use of PFS reduces the number of steps required for dose preparation and handling since the physician does not need to withdraw the product from a vial (36). PFS also provides an opportunity to customize key features such as syringe size and hold-up volume to achieve improved syringeability and dose accuracy. Pre-filled syringes are assembled by filling the drug into a syringe barrel followed by sealing of the barrel with a rubber stopper. The stopper is further attached to a piston rod that is used to activate the stopper movement during dose preparation and injection. Syringe barrels made of glass are commonly used for IVT delivery. Developers must understand potential extractables and leachables from the PFS and characterize their interaction with the drug product (40). Although a complete review of extractables and leachables is out of scope for this manuscript, we would like to highlight aspects highly relevant to biologics for IVT administration.

Typically, it is necessary to provide lubrication between the rubber stopper and the glass surface to achieve acceptable force required to activate the stopper (breaking force) and to move the stopper along the barrel to deliver the drug (gliding force). Syringe manufacturers have used the application of silicone oil on both syringe barrels and stoppers to achieve the desired lubrication. Several studies have been published that provide an overview and comparison of various barrel siliconization processes using either medical grade silicone oil, polydimethylsiloxane (PDMS) for spray siliconization or baked-on siliconization (a process comprised of coating the barrel with silicone oil-in-water emulsions followed by baking at 120–350°C and subsequently washing the barrels to remove any non-fixed silicone to provide lubrication (41). Cross-linked siliconization process has also been recently reported and interestingly has shown to decrease the amount of leachable silicone oil while maintaining the functionality (42,43).

Development strategy for a glass PFS, lubricated with silicone oil, for IVT administration of a biologic should, at a minimum, focus on establishing compatibility of drug product with the container closure, including: characterization of the effect of silicone oil extractables on the quality and efficacy of the drug, characterization of leachable silicone oil over the shelf-life of the product, and contribution of leached silicone oil to particulate matter in the drug product and when injected into the vitreous cavity. Contribution of silicone oil to particle generation and aggregation in protein drug products has been widely reported (44–46). Proteins may adsorb to the oil-water interface (47) or silicone oil leached from the barrel into the protein formulation may result in aggregation (44–46). The siliconization method was shown to not have an impact on particle concentration in the absence of an air bubble in the filled syringes but may impact the standards for acceptable particulate matter in ophthalmic injections which are described in pharmacopoeial guidance such as USP <789> or Ph Eur 5.7.1. Due to a stringent criterion compared to other parenteral injections, the contribution of leached silicone oil may be more significant in IVT products.

A glass PFS that does not require siliconization has the potential to not only reduce the particulate matter but also reduce the destabilization of biologics sensitive to silicone oil. One such system was described in USPT10,471,212 and USPT8,722,178. The system comprises of a non-siliconized glass barrel and a stopper that is coated with a barrier layer such as PTFE to provide the necessary lubrication. Although only glass PFSs have been commercially available, several non-glass PFSs have been proposed in published literature and patents (22).USPT20170232199 describes a plastic syringe made of cycloolefin polymer or cycloolefin copolymer that is silicone-free and is suitable for IVT administration. Higher gas permeability of plastic makes development of non-glass syringes challenging due to the requirement of terminal sterilization of ophthalmic products. Correspondingly, appropriate risk assessments and studies should be performed.

Another potential leachable from pre-filled syringes that warrants characterization is tungsten. Tungsten pins are routinely used during syringe forming process, and residual tungsten can potentially leach into the product. Although the syringe manufacturing process involves washing steps, they may not eliminate residual tungsten. Small amounts of residual tungsten may be sufficient to cause metal-catalyzed protein oxidation. Residual tungsten may also lead to aggregation and particle formation (48,49).

## TERMINAL STERILIZATION OF OPHTHALMIC PRODUCTS

### Requirements for Terminal Sterilization for IVT PFS Drug Products

Sterility of ophthalmic products can be achieved by aseptic processing and/or terminal sterilization. Although discussions on aseptic processing are out of scope for this manuscript, guidance documents such as ISO 13408-1:2008 provide the requirements for aseptic processing of healthcare products (50). Terminal sterilization is performed for medical devices and pre-filled syringes among other drug product configurations for the treatment of several disease indications. Sustained-release ocular drug products administered using a medical device or pre-filled syringe will also require terminal sterilization. Terminal sterilization is defined as a “process whereby product is sterilized within its sterile barrier system” (51) (ISO/TS 11139:2006). Terminal sterilization is a critical unit operation and is carried out towards the end of the product manufacturing process. Pre-filled syringe drug products for IVT are terminally sterilized and packaged to maintain sterility and provide a sterility assurance level (SAL) of 10^−6^ or one non-sterile unit in 1,000,000 units throughout the shelf-life of the product. The SAL is governed by industry guidance for drug products termed “sterile” (52) (ANSI/AAMI ST67:2003).

Effective terminal sterilization requires high degree of process control which ensures product quality and appropriate SAL for a given drug product. Therefore, process validation of terminal sterilization is critical, and manufacturers often perform extensive studies and validation campaigns to ensure product sterility and impact of terminal sterilization on the drug product. The reader is encouraged to refer to the following references for additional regulatory requirements for terminal sterilization of medical devices:

1. International Organization for Standardization 11040-4:2015, Prefilled syringes–Part 4: glass barrels for injectables and sterilized sub-assembled syringes ready for filling
2. International Organization for Standardization 11040-6:2012, Prefilled syringes–Part 6: plastic barrels for injectables
3. ANSI/AAMI/ISO 11607:2006, Packaging for terminally sterilized medical devices
4. USP 27:2004, Sterility, Biocompatibility, Biological Tests and Assays, Bacterial Endotoxin Test (LAL), Pyrogen Test (USP Rabbit Test), or other applicable tests related to the drug/biological product and delivery of the drug/biological product
5. AAMI/ANSI/ISO 11737-1:2006, Sterilization of medical devices-microbiological methods-Part 1: Determination of the population of microorganisms on products
6. USP-NF <1222>, Terminally sterilized Pharmaceutical Products-Parametric Release

#### Vapor-Phase Hydrogen Peroxide

Vaporized hydrogen peroxide is a highly effective sanitizing agent used in aseptic manufacturing facilities against spores, bacteria, and viruses. Hydrogen peroxide is an oxidizing agent which targets lipids, nucleic acids, and proteins within the microbes. Interestingly, the mechanism of action for the liquid form is different from the gaseous hydrogen peroxide. The gaseous form of hydrogen peroxide has been shown to inactivate pyrons more efficiently than liquid hydrogen peroxide (53).

Widespread implementation of terminal sterilization using vapor-phase hydrogen peroxide (VHP) is still in its infancy primarily driven by limitations such as incompatibility with cellulosic material, penetration of the sterilant, and variance in microbial inactivation kinetics. However, additional studies with advanced technologies such as flow cytometry and genetic sequencing on the resistant microbes would help determine the effectiveness of vaporized hydrogen peroxide sterilization and enable appropriate process validation (54). Although a very effective sanitizing agent, the use of vaporized hydrogen peroxide is associated with the risk of residual hydrogen peroxide in drug products that may lead to oxidation of biologic drug products. During development, scientists must pay attention to the permeability of selected container closure components to VHP and characterize for its ingress into the product and any associated quality implications.

#### Ethylene Oxide Sterilization

Ethylene oxide, radiation, and steam sterilization are among the most common type of sterilization methods in the pharmaceutical/biotech industry. Ethylene oxide is a highly reactive cyclic ether which is a gas at room temperature and is liquified for use as a sterilant. Ethylene oxide causes alkylation of the amine groups within the microbial DNA which leads to microbial death. Although ethylene oxide is extremely effective and widely used in the manufacturing industry for sterilization of medical devices and pharmaceutical/biotech drug products, ethylene oxide (EO) is a toxic gas with safety implications to the staff, environment, and patients if handled inappropriately (55).

Since ethylene oxide is widely used for external surface sterilization in manufacturing, the FDA requires submission of significant manufacturing control data and documentation to demonstrate sterility and acceptable quality of the drug product for commercial products. Briefly, the filled/finished product is loaded on to pellets and exposed to a validated combination of humidity, ethylene oxide gas, temperature, and time. Deep vacuum cycles aid in driving humidity and ethylene oxide into palletized product. Following the exposure of EO to the pelletized product, a validated in-chamber vacuum purge process or a post-sterilization aeration process is applied to achieve EO levels below permissible exposure limits (56).

One important quality attribute is the residual ethylene oxide present during manufacturing of biologics that can alter drug product quality during storage of commercial products. Therefore, the FDA also requires quantitative data that demonstrates acceptable residual levels of ethylene oxide that does not alter the quality attributes of the drug product.

In all cases where terminal sterilization is being evaluated with ethylene oxide, it is essential to determine the container-closure integrity; as for liquid products, ingress of ethylene oxide into the aqueous environment would lead to formation of ethylene glycol in the drug product. Another common impurity is the presence of ethylene chlorohydrin which may form when ethylene oxide comes in contact with free chloride ions present in glass and plastic (57).

#### Nitrogen Dioxide Sterilization

Nitrogen dioxide sterilization of external surfaces has emerged as an alternative to the ethylene oxide sterilization process due to its ease of handling and a sterility assurance level similar to ethylene oxide. Nitric oxide is a reddish-brown gas with a boiling point of 21°C at sea level which can be introduced rapidly into packages in the sterilizing chamber with little to no vacuum. A potential advantage of no vacuum is the reduction in risk of stopper movement in pre-filled syringes under vacuum. Sterilization and aeration processes can be carried out at room temperature or lower; this is an advantage as compared to ethylene oxide and hydrogen peroxide sterilization processes. Furthermore, the concentrations of gas required to achieve terminal sterilization are relatively low (1–2%). Another significant advantage as compared to ethylene oxide and hydrogen peroxide sterilization is the time required to run a typical sterilization cycle which ranges from 2 to 3 h as compared to days for ethylene oxide sterilization cycles (58,59). Nitrogen dioxide inactivates all forms of microorganisms, including bacteria, bacterial spores, fungi, fungal spores, and viruses. The mechanism of microbial kill is primarily through single-stranded breaks in the DNA which increase with increasing nitrogen dioxide concentration (60). Materials that are compatable/non-compatible for sterilization using Nitrogen dioxide are listed in Table IV.

**Table IV:** Material Compatibility for Nitrogen Dioxide Sterilization Process

Compatible	Not compatible
Stainless steel, polyethylene, polyetherimide, anodized aluminum, polypropylene, polycarbonate, gold (plating), PET/PETG, cyclic olefins, glass/ ceramic, polystyrene, PVC^a^, fluoropolymers, polysulfones, silicone^a^, Viton (gaskets), Hypalon	Polyurethane, thermoplastic elastomers (TPE), nylon, polyester, polyolefin, Delrin (polyacetal), PSU, PEI, cellulose-based (some paper), polyester or styrene label stock

^a^Depending on the material grade

Not an exhaustive list

A head-to-head sterilization study was conducted on syringe tubs prior to filling operation where biological indicators (BI) are placed at various locations on the syringe tub and Tyvek bags. The nitrogen dioxide sterilization process was carried out for 15 min as opposed to the vaporized hydrogen peroxide process carried out for 43 min. Results demonstrated that the nitrogen dioxide cycle was consistent and lethal to all BI across several syringe tubs placed at different locations in the sterilizing chamber. However, the VH sterilization cycle was not very effective in its lethality against the BI across several syringe tubs (61). Another study demonstrated the ability of the nitrogen dioxide gas to be used as a surface sterilant for pre-filled syringes. The study described 1-mL glass syringes that were filled with water for injection and exposed to the nitrogen dioxide sterilization cycle. The authors demonstrate no ingress of nitrogen dioxide (assay limit < 0.024 ppm) through the syringes. Since NO_2_ converts to NO_3_^-^, the detection of NO_3_^-^ can be performed by colorimetric assay as a release test (62).

Although several studies have demonstrated the advantages of using nitrogen dioxide, it is a relatively newer technology which would benefit from additional studies and white papers/publications in collaboration with industry and academia.

### Challenges with Terminal Sterilization Using Oxidizing Agents

#### Impact of Residual Sterilizing Oxidant on Container Closure Systems

Studies have demonstrated the impact of vaporized hydrogen peroxide sanitization within isolators on platinum cured silicone tubing, glass vials, syringes, and stoppers. It is important to understand the interaction of container closure systems and commonly used components in the manufacturing process with the sterilizing agent to ensure the sterilizing agents do not have a detrimental effect on product quality. For example, silicone tubing had decreased propensity to adsorb vaporized hydrogen peroxide as compared to gamma irradiated silicone tubing. Glass vials of various sizes when exposed to varying levels of VHP (50 to 500 ppb) demonstrated a correlation between VHP concentration in the isolator and adsorbed VHP within the vial albeit with high variability among replicate vials. The variability was not attributed to isolator air flow but rather the varying rate of VHP diffusion into the vials. Stoppers used in drug products are generally coated with hydrophobic fluoro-polymer to minimize drug-stopper interaction; exposure of these coated stoppers to VHP was observed to have negligible levels of VHP adsorption. Empty 1-mL syringes were exposed to 500 ppb VHP for 24 h and were observed to have negligible amount of hydrogen peroxide adsorption in the inner surfaces of the syringe. Inner surfaces of prefilled syringes are coated with silicone oil for ease of injection which makes the inner surfaces hydrophobic thereby reducing the amount of hydrogen peroxide adsorbed on the surface. In general, it was observed that there was a correlation between surface hydrophilicity and the amount of hydrogen peroxide adsorbed. Therefore, unit operations that reduce the hydrophilicity of surfaces (reduced water) would lead to reduced hydrogen peroxide uptake (63).

#### Impact of Ingress of Sterilizing Oxidant on Drug Product Quality

Undesired ingress of oxidizing sterilizing agent into the drug product during terminal sterilization can have significant safety and efficacy concerns for biologics. Several amino acid residues such as methionine, cysteine, histidine, and tryptophan have been identified as “hot spots” for oxidation events following exposure to hydrogen peroxide (64). To assess the impact of VHP ingress, hydrogen peroxide spiking studies are commonly performed during drug product development to determine the rate and extent of protein degradation. Residual hydrogen peroxide impacts not only liquid protein formulations but also drug products that undergo lyophilization. Cheng *et al.* demonstrated that when protein formulations are spiked with 5 ppm of hydrogen peroxide prior to lyophilization, an average of 94.1% of the spiked hydrogen peroxide was removed during lyophilization (56). Oxidation occurred during lyophlization and even when the formulation is frozen. Furthermore, oxidized proteins were prone to aggregation during the lyophilization process (64). Similar to vaporized hydrogen peroxide, detrimental effects of ethylene oxide have been reported in the literature; significant degradation of human serum albumin and pegylated granulocyte-colony stimulating factor have been reported (66). Therefore, undesired ingress of ethylene oxide into the drug product has been demonstrated to be detrimental to the drug product depending on protein oxidation potential, formulation characteristics, and primary container material, all of which have the potential to for unwanted clinical consequences (67). Eisner et al. demonstrated the importance of sample handling while performing analysis for hydrogen peroxide in drug product. Degradation of hydrogen peroxide was observed to be faster at − 20°C when compared to 2–8 °C when antibody formulations were spiked with hydrogen peroxide, while hydrogen peroxide was most stable in antibody formulation when stored at − 70°C (65)

London *et al.* demonstrated that external surface terminal sterilization with ethylene oxide on ranibizumab pre-filled syringes did not have any negative impact on ranibizumab potency, concentration, and stability when incubated at 2–8°C for ≤ 3 years (68).

Funatsu *et al.* demonstrated the presence of residual ethylene oxide on empty cycloolefin polymer barrels which were sterilized using ethylene oxide (ISO 11135:2014 standards) prior to filling of the formulation (58). In the study, the authors demonstrate significant degradation of human serum albumin at cysteine and methionine residues with ethylene oxide concentrations as low as 34 μg/syringe. It is important to note that residual ethylene oxide levels for EO-sterilized medical device based on the ISO 10993-724 standard is 4000 mg/syringe (67). Therefore, it must be noted that although regulatory requirements may be met for residual levels, the levels required for maintaining drug product quality may be more stringent.

These considerations for biologic drug products are not unique to a specific administration route, and biologics intended for IVT administration also need to be carefully monitored for protein degradation events to ensure product safety and efficacy.

## IVT PRODUCTS THAT EXTEND DURATION OF IVT INJECTIONS IN THE CLINIC

Reducing dosing frequency of IVT injections would be a significant improvement in the patient’s quality-of-life suffering from chronic indications such as wet age-related macular degeneration and diabetic retinopathy where frequent IVT injections are administered.

Intraocular delivery devices have been established and approved by the FDA for small-molecule therapeutics; however, there are no approved devices delivering biologics. Several companies are focused on developing novel devices and delivery systems for IVT sustained release of biologics which reduce frequency of injection for biologics.

There are several considerations for the development for sustained release devices which include factors such as selection of biodegradable or non-biodegradable polymer, polymer back bone chemistry, immunogenicity of the polymer system, stability of the molecule, size of the device, site of implantation, implantation procedure, repeat dosing and/or device refill procedure, and time interval between repeat dosing (69).

Several academic and industry publications have focused on biodegradable polymers such as poly(lactic-co-glycolic acid), polycaprolactone, and proprietary polymer blends that degrade at specific rates for release of active ingredient in the vitreous. However, only a handful of approaches are in clinical development as therapies for reducing dosing frequency while maintaining therapeutic efficacy (Table V).

**Table V:** Clinical Investigation of Therapeutics for Reduced IVT Dosing Frequency

Technology	Sponsor	Mechanism of action	Latest phase
Bioresorbable hydrogel containing tyrosine kinase inhibitor	Ocular Therapeutix	Tyrosine kinase inhibitor	Phase I
Anti-VEGF mAb conjugated to a phosphorylcholine-based biopolymer	Kodiak Sciences	Anti-VEGF	Phase III
Bispecific antibody angiopoietin-2/VEGF-A (Faricimab)	Roche	Anti-VEGF Anti-Ang-2	Phase III
Sunitinib malate microparticle depot	Graybug Vision	Anti-VEGF	Phase IIa/Phase IIb
ADVM-022 IVT gene therapy	Adverum Biotechnologies	Anti-VEGF	Phase II
Fusion protein aflibercept—high dose	Regeneron Pharmaceuticals	Anti-VEGF	Phase II/III
Port delivery system	Roche	Anti-VEGF	Phase III

## CONCLUSION

IVT injections are standard of care for treating various intraocular diseases. The clinical procedure for successfully injecting drugs in the intravitreal space has been studied and reported on extensively by retina specialists, academicians, and industry scientists. The procedure also requires careful selection of administration components such as syringes and needles. In recent years, there has been a specialized focus on improving intravitreal injection devices and products. Examples include improvements in needle designs that enhance injectability and reduce complications such as vitreal reflux for chronic diseases such as diabetic macular edema and wet-AMD. Improvements in syringe designs such as the development of silicone-free syringes and needle bevel designs can further improve existing drug products specific for intravitreal administration. Maintaining accuracy and precision while injecting small volumes is challenging, and it is important to understand the capability of the administration components during drug product development.

In addition to intravitreal injections, other injection routes such as suprachoroidal and sub-retinal injections have demonstrated promise in delivering therapeutics to the intended site of action within the ocular tissues. Microneedles are one such devices that can enable injection of therapeutics into ocular tissues while being minimally invasive as compared to intravitreal injections.

Furthermore, the latest generation of therapeutics which include cell and gene therapies may bring a new paradigm for ophthalmic drug product development. Depending on the intended site of action, development of specialized devices, materials, formulations, and manufacturing processes may be warranted which meet the criteria for safety and efficacy as per regulatory guidelines.
